# Short-Time Magnetron Sputtering for the Development of Carbon–Palladium Nanocomposites

**DOI:** 10.3390/nano14020164

**Published:** 2024-01-12

**Authors:** Florian Knabl, Nikolaos Kostoglou, Velislava Terziyska, Steven Hinder, Mark Baker, Etienne Bousser, Claus Rebholz, Christian Mitterer

**Affiliations:** 1 Department of Materials Science, Montanuniversitӓt Leoben, 8700 Leoben, Austria; velislava.terziyska@unileoben.ac.at (V.T.); claus@ucy.ac.cy (C.R.); christian.mitterer@unileoben.ac.at (C.M.); 2 Department of Mechanical Engineering Sciences, University of Surrey, Guildford GU2 7XH, UK; s.hinder@surrey.ac.uk (S.H.); m.baker@surrey.ac.uk (M.B.); 3 Centre for Characterization and Microscopy of Materials (CM)2, Polytechnique Montréal, Montréal, QC H3T 1J4, Canada; etienne-2.bousser@polymtl.ca; 4 Department of Mechanical and Manufacturing Engineering, University of Cyprus, Nicosia 1678, Cyprus

**Keywords:** physical vapor deposition, magnetron sputtering, surface functionalization, palladium, activated carbon cloth, metal–carbon composites

## Abstract

In recent nanomaterials research, combining nanoporous carbons with metallic nanoparticles, like palladium (Pd), has emerged as a focus due to their potential in energy, environmental and biomedical fields. This study presents a novel approach for synthesizing Pd-decorated carbons using magnetron sputter deposition. This method allows for the functionalization of nanoporous carbon surfaces with Pd nano-sized islands, creating metal–carbon nanocomposites through brief deposition times of up to 15 s. The present research utilized direct current magnetron sputtering to deposit Pd islands on a flexible activated carbon cloth substrate. The surface chemistry, microstructure, morphology and pore structure were analyzed using a variety of material characterization techniques, including X-ray photoelectron spectroscopy, X-ray diffraction, Raman spectroscopy, gas sorption analysis and scanning electron microscopy. The results showed Pd islands of varying sizes distributed across the cloth’s carbon fibers, achieving high-purity surface modifications without the use of chemicals. The synthesis method preserves the nanoporous structure of the carbon cloth substrate while adding functional Pd islands, which could be potentially useful in emerging fields like hydrogen storage, fuel cells and biosensors. This approach demonstrates the possibility of creating high-quality metal–carbon composites using a simple, clean and economical method, expanding the possibilities for future nanomaterial-based applications.

## 1. Introduction

Recent research in nanoscience and nanomaterials has increasingly focused on creating efficient and cost-effective nanoscale composites. In particular, nanocomposites composed of nanostructured and/or nanoporous carbons, such as activated carbons, templated mesoporous carbons, carbon nanotubes, few-layer graphene and others and metallic and/or inorganic nano-sized particles, clusters or thin films, have gained significant attention for emerging energy [1,2], environmental [3] and biomedical applications [4]. This is due to the attractive properties of the individual phases and also because of potential synergistic effects [5].

In this respect, palladium (Pd), a member of the platinum group metal family, has shown promising catalytic activity with a variety of uses, e.g., as an essential component in catalytic converters to reduce harmful emissions in the transport sector. Switching from bulk Pd to nanostructured Pd can offer numerous advantages, e.g., reduced use of scarce and costly resources or resulting from changes in physical and chemical properties, largely influenced by the higher surface area-to-volume ratio. Pd-based nanomaterials, like Pd nanoparticles (NPs), are used in various emerging fields, including biomedicine for antibacterial activity and anticancer therapy [6]. In energy sectors, they find roles in fuel cells [7] and hydrogen storage [8,9]. Additionally, these nanomaterials are used in electronics for sensor applications [10]. 

Pd NPs can be synthesized via a plethora of physical, chemical and biological methods. Physical methods include magnetron sputter inert gas condensation [11,12,13] and laser ablation [14], while chemical methods include electrochemical deposition and wet chemistry (e.g., sol–gel methods) [10]. In the field of biology, naturally occurring biomolecules or metabolites from different organisms are used, with a recent summary given by Joudeh et al. [10]. Growth of Pd thin films was, to the best of the authors’ knowledge, already studied in 1972 by Sing and Murr [15] and the interest increased significantly with the development of magnetron sputtering [16]. A first study on the morphology of sputter-coated Cu, W and Pd was reported by Haas and Birringer in 1992 [17]. 

Referring to possible substrates, nanoporous carbon-based fabrics, cloths and felts composed of activated carbon fibers have been proposed as promising hosts for NPs, mainly because of their lightweight nature, increased mechanical integrity/flexibility, good thermo-chemical stability, large surface area and high porosity [1,2,3,18]. These types of materials offer additional benefits in terms of practicality and ease of handling compared to others (e.g., fine/volatile powders), thus being more likely to be employed as functional components in devices and systems where mechanical robustness plays an important role. In this respect, Pd-decorated activated carbon cloths have been suggested in the literature to be employed in high-tech applications such as sorbents for hydrogen storage [19,20], cathodes for the oxygen reduction reaction [21], biosensors [22] as well as hydrogen sensing [23,24].

Within this work, it is shown for the first time that conventional magnetron sputter deposition processes may be utilized for the functionalization of nanoporous carbon cloths with Pd nano-sized islands to develop metal–carbon composite materials through brief deposition times (in a matter of seconds). Pd island-decorated carbon cloths were synthesized by varying the deposition times. Under the applied conditions and with the expected stronger interaction between Pd–Pd compared to Pd–C atoms, three-dimensional island growth can be expected to dominate (Volmer–Weber growth) [25]. Sample morphology, surface chemistry, microstructure and pore structure of the synthesized Pd-island-decorated carbon cloths were assessed using a series of materials characterization methods, including X-ray photoelectron spectroscopy (XPS), X-ray diffraction (XRD), Raman spectroscopy, gas sorption analysis (N_2_@77 K), and scanning electron microscopy (SEM). These studies revealed coverage with Pd islands of varying rates and sizes “sprinkled” on the surface of the carbon fibers. The proposed approach combines the key advantages of a clean, physical synthesis procedure, free of chemical precursors with high-purity surface modifications, while avoiding the high costs and lack of accessibility associated with dedicated magnetron sputter inert gas condensation systems used for gas-phase NP synthesis. The larger NP size range synthesized by this method is not readily achieved with magnetron sputter inert gas condensation, as the latter usually yields comparatively smaller NPs (i.e., <20 nm).

## 2. Materials and Methods

### 2.1. Synthesis of Activated Carbon Cloth Substrates

For this study, a woven activated carbon cloth (ACC) was employed as the substrate material. Viscose rayon cloth (VRC) was used as the precursor material for the ACC. At first, the VRC was impregnated into a mixture of zinc chloride (ZnCl_2_) and ammonium chloride (NH_4_Cl) aqueous solution, with remnants of those chemical compounds confirmed via XPS during subsequent characterization steps. The soaked VRC sample was then air-dried and carbonized up to 630 °C under N_2_ atmosphere. A final activation step using CO_2_ was performed up to 930 °C. The ACC obtained after additional washing and drying steps retains the cloth structure of the VRC and is thus both flexible and mechanically stable. The ACC substrate and its possible applications were previously presented by our research group [26] and more details about the synthesis procedure can be found in earlier studies by Babic and co-workers [27,28]. The ACC substrates used within this study were cut to square pieces with approximate dimensions of 14 mm × 14 mm, with each piece weighing around 100 mg.

### 2.2. Synthesis of Carbon–Palladium Nanocomposites

The deposition experiments were conducted using a custom-made laboratory-scale unbalanced direct current (DC) magnetron sputtering system, already described in detail in earlier studies [29]. This system is equipped with three 50.8 mm diameter unbalanced AJA A320-XP magnetrons focused towards a rotating substrate holder. While two of these magnetrons were not used for this work, a Pd target (99.99% purity, 6 mm in thickness), supplied by Kurt J. Lesker (Jefferson Hills, PA, USA), was employed for sputtering using the third magnetron. Prior to deposition, the vacuum chamber was evacuated to a base pressure below 2 × 10^−5^ mbar. Pumping was carried out using a turbomolecular pump backed by a rotary vane pump. The substrates were positioned on a rotating substrate holder within the deposition system and rotated at 50 rpm during etching and deposition. Samples were kept under vacuum for 2 h prior to etching with no additional heat being applied during both etching and deposition. Substrate etching was carried out for 60 s with a closed shutter using an etching current of 0.1 A, an asymmetrically pulsed DC voltage of −500 V with 50 kHz and an Argon (Ar) gas flow of 200 sccm (corresponding to a pressure of 3 × 10^−2^ mbar) in order to clean the substrate surface. Simultaneously, the Pd target underwent sputter cleaning with closed shutters. Prior to the deposition, the Pd target was pre-sputtered for 5 min to ensure a clean target surface as well as stable sputtering conditions. 

The deposition process was conducted in constant-current mode set at 0.1 A, with an Ar gas flow of 30 sccm, corresponding to a pressure of 5 × 10^−3^ mbar, and a target-to-substrate distance of 45 mm. Process parameters were determined in preliminary, unpublished studies and chosen to ensure a low deposition rate while maintaining a stable plasma throughout the deposition. During deposition, no external heating was employed, and the substrates were maintained at a floating potential as no bias voltage was applied. The deposition time was controlled using a shutter. Three samples were prepared with varying deposition times of 5, 10 and 15 s on each side of the ACC substrates. These samples were subsequently labeled as *Pd-ACC-5s*, *Pd-ACC-10s* and *Pd-ACC-15s*, respectively, for the purpose of this work, with the pristine/uncoated reference sample labeled as *ACC*.

### 2.3. Characterization Methods

XPS measurements were conducted with a Thermo Scientific Theta Probe system (Waltham, MA, USA), which uses a monochromated Al Kα X-ray source (1486.6 eV photon energy) and a ~400 μm in radius X-ray spot. Wide-scan survey spectra and high-resolution core level spectra for the different components were collected with a pass energy of 300 eV and 50 eV, respectively. To compensate for potential charging effects that might occur during the measurement process, all acquired spectra were charge referenced to the C1s peak at 284.5 eV (sp^2^ hybridized carbon). For the quantitative analysis of the chemical compositions, the high-resolution core level spectra were adjusted by instrument-modified Wagner sensitivity factors, upon applying a non-linear Shirley background subtraction.

XRD studies were carried out using a Bruker-AXS D8 Advance diffractometer (Billerica, MA, USA), which utilizes Cu Kα radiation (~0.154 nm wavelength), operating at a 40 kV voltage and a 40 mA current. Diffraction patterns were obtained using the Bragg–Brentano configuration with a continuous scan speed between the diffraction angles 2θ 10–90°, advancing at a 0.01° step width and a 0.5°/min scanning rate.

Raman spectra were recorded using a Jobin–Yvon LABRAM confocal spectrometer (Lille, France), which is equipped with a frequency-doubled Nd-YAG laser (532.2 nm emission wavelength) and a Peltier-cooled slow-scan charge-coupled device matrix detector. The laser beam was focused using an Olympus BX 40 microscope, fitted with a ×50 long-working distance objective lens, using a 0.1 mW/μm^2^ power density and a 1.5 cm^−1^ spatial resolution. The spectroscopic data were collected in the wavenumber region of 500–2000 cm^−1^.

Nitrogen (N_2_) gas adsorption and desorption isotherms were collected at 77 K (−196 °C) with an Anton Paar QuantaTec Autosorb-iQ^3^ manometric gas sorption analyzer (Boynton Beach, FL, USA) by employing ultra-pure (99.999%) N_2_ gas as adsorbate, ultra-pure (99.999%) helium (He) gas for void volume calculations and liquid N_2_ as cryogen. Prior to the tests, cloth samples of ~40 mg were degassed under vacuum (10^−6^ mbar) at 250 °C for 24 h to remove physisorbed surface species and make the nanopore structure more accessible. The specific surface area was calculated by applying the multi-point Brunauer-Emmett-Teller (BET) method, following the BET consistency criteria (ISO 9277:2022) in the adsorption data for relative pressures (P/P_0_) between 0.01 and 0.05. The specific pore volume for pores smaller than 57 nm was estimated using the single-point Gurvich rule at P/P_0_ ~0.96. The micropore surface area and micropore volume values were determined by applying the Carbon Black statistical thickness (t-plot) method in the P/P_0_ range 0.2–0.5. The pore size/width distribution and mean pore size/width were deduced using the quenched solid density functional theory (QSDFT) method based on the N_2_-carbon equilibrium transition kernel at 77.35 K for slit-shaped pores.

SEM investigations were performed using a Zeiss Merlin ultra-high-resolution field emission gun scanning electron microscope (Oberkochen, Germany) equipped with a Gemini II column. Secondary electron images using an InLens detector and back-scattered electron images were acquired at 4 kV acceleration voltage. The elemental maps for carbon and palladium were collected with two parallel integrated Oxford Instruments XMAX 150 mm^2^ energy dispersive X-ray spectroscopy (EDX) detectors using the same acceleration voltage.

## 3. Results & Discussion

### 3.1. Surface Chemistry and Elemental Composition

**Figure 1:**
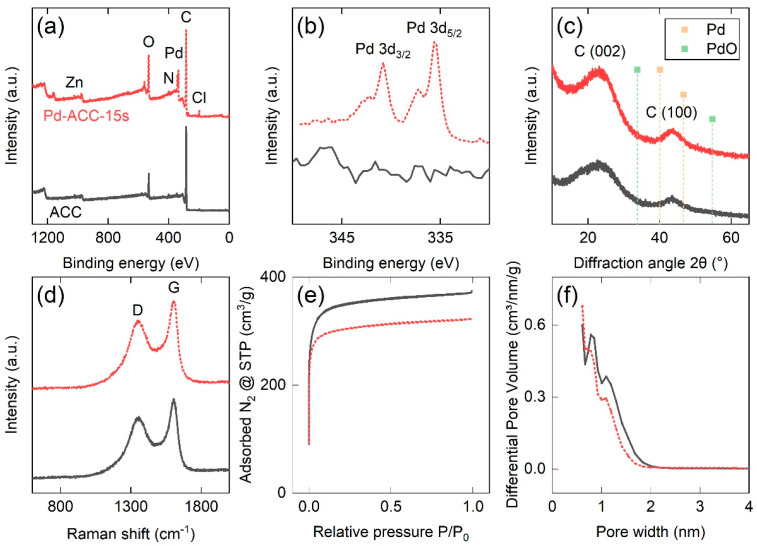
(**a**) Wide-scan X-ray photoelectron survey spectra and (**b**) high-resolution Pd spectra, (**c**) X-ray diffractograms, (**d**) Raman spectra, (**e**) N_2_ adsorption and desorption isotherms recorded at 77 K and (**f**) pore size distribution analysis derived by the quenched solid density functional theory method for slit-shaped pores for the pure *ACC* and *Pd-ACC-15s* samples.

In the first step of the investigation, XPS measurements of all samples (pure and Pd-decorated) were conducted to determine the surface chemical composition. The XPS survey spectra revealed that all samples contained the elements of C, O, N, as well as small traces of Zn and Cl, with the latter stemming from the carbon cloth synthesis process (i.e., impregnation into an aqueous mixture of NH_4_Cl and ZnCl_2_ prior to carbonization). This is consistent with earlier reports on the chemical composition of the carbon cloth substrate [26]. The average Pd content was determined as 0, 0.8, 1.8 and 2.6 at % for the pure *ACC* reference and the subsequent samples ranging from 5 s to 15 s of deposition time, thus revealing maximum Pd content at 15 s. To facilitate characterization, subsequent investigations were focused on this particular Pd-rich sample. A comparison of the XPS survey spectra of pure *ACC* and *Pd-ACC-15s* samples is given in Figure 1a, revealing a distinct Pd peak for the Pd-decorated sample, among multiple other elements. High-resolution spectra in the binding energy range of Pd are depicted in Figure 1b, showing the presence of a peak doublet of the Pd 3d orbital in the *Pd-ACC-15s* sample and a lack thereof in the pure *ACC* sample. The Pd 3d_5/2_ spectrum in Figure 1b shows the presence of two peaks at binding energies of 335.6 eV and 337.5 eV, corresponding to Pd metal and its native oxide, respectively. These binding energies are generally in good agreement with those presented in other studies [30,31,32], but shifted to a slightly higher binding energy. Such positive electron binding energy shifts have been previously reported for nano-sized particles on surfaces, including Pd on amorphous carbon [33].

### 3.2. Microstructural Characterization

Subsequent structural characterization studies were carried out using XRD with the respective diffractograms shown in Figure 1c. For both samples, a broad peak roughly centered at 2θ ~23° and with a full width at half maximum of ~15° was observed. An additional peak of much lower intensity was recorded between 43° and 44°. These peaks are commonly reported in carbons with a low structural order, such as turbostratic carbons, and are related to the (002) and (100) reflections of graphite, respectively [26]. Even for the highest Pd content among the investigated samples, no obvious structural changes were visible from the X-ray diffractograms before and after Pd deposition. In addition, no other peaks were recorded at positions related to Pd or corresponding Pd oxides, being in good agreement with the obtained low Pd content determined by XPS and/or indicating amorphous growth. Furthermore, Raman spectroscopy studies (Figure 1d) revealed distinct defect-activated D and graphitic G bands of identical appearance in the exact same Raman shifts (i.e., ~1350 cm^−1^ and ~1605 cm^−1^, respectively) and with comparable D/G intensity ratios (i.e., 0.78 vs. 0.77, respectively), thus implying that no structural changes are caused in the carbon cloth substrate due to the Pd deposition process.

### 3.3. Pore Structure Properties

A follow up investigation in terms of gas sorption analysis was carried out by recording N_2_ adsorption and desorption isotherms at 77 K, as depicted in Figure 1e. Both materials demonstrate characteristic fully reversible Type I isotherm shapes according to the classification of the International Union of Pure and Applied Chemistry (IUPAC) [34], associated with the dominant presence of micropores (i.e., pore sizes/widths below 2 nm). Both isotherms indicate a significant and comparable uptake of N_2_ already at very low relative pressures (P/P_0_ < 10^−5^) due to enhanced gas–solid interactions in sub-nanometer pores, followed by the formation of a clear saturation plateau due the complete filling of the accessible micropore volume [34]. The overall N_2_ uptake at higher relative pressures is reduced by ~12% for the Pd-decorated sample. This might be explained by a limited pore blocking effect caused by the Pd decoration procedure, a phenomenon commonly reported in the literature [35,36]. The pore size/width distribution was extracted from the desorption branch of the isotherm, as seen in Figure 1f, and revealed a trimodal pore size distribution with the strongest contributions stemming from pores with widths of ~0.6, ~0.8 and ~1.1 nm, thus further confirming the presence of both ultra-micropores (<0.7 nm) and super-micropores (i.e., 0.7–2 nm), as classified by the IUPAC. 

Table 1 highlights the changes in the pore structure features, including specific surface area, specific pore volume, micropore surface area/volume and mean pore size/width, before and after the 15 s sputtering procedure. The BET surface area of the pure *ACC* (1365 m^2^/g) decreased by ~12% upon Pd deposition, while similar trends were also found for the change in the total pore volume, micropore surface area and micropore volume. The d_50_ value, corresponding to a pore width of the 50% of the QSDFT-derived pore volume, slightly shifted from 0.77 to 0.67 nm upon Pd deposition. This implies that most of the change in pore volume occurs at larger pore widths while smaller pores are not significantly affected. This is also supported by the fact that the percentage of microporosity (i.e., ratio of micropore volume to total pore volume) remains the same after deposition, i.e., ~88% for both pure *ACC* and *Pd-ACC-15s* samples. The findings of the gas sorption analysis confirm that the nanopore structure of the Pd-decorated sample is not significantly affected by the magnetron sputter deposition process, despite the small reduction of the available surface area and pore volume.

### 3.4. Surface Morphology

**Figure 2:**
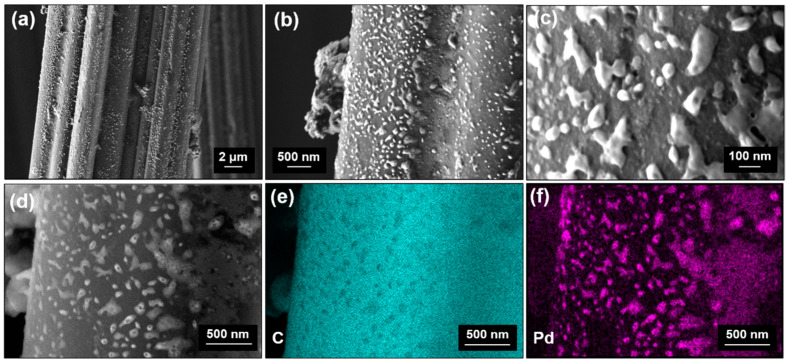
(**a**–**c**) In-lens field emission scanning electron microscopy micrographs (topography contrast) in different magnifications and (**d**) backscatter electron micrograph (chemical contrast) for the *Pd-ACC-15s* sample. Energy dispersive X-ray spectroscopy mappings were recorded for (**e**) carbon and (**f**) palladium elements.

Finally, high-resolution SEM and EDX investigations were carried out. A representative collection of the SEM images and elemental maps for the *Pd-ACC-15s* sample is given in Figure 2. In detail, Figure 2a shows an individual carbon fiber of the cloth, showing a corrugated circumference with a width/diameter of roughly 10 µm. Higher magnifications (Figure 2b,c) reveal the development of particles appearing brighter in contrast on the dark carbon fiber surface. The morphology may be described as nano-sized islands or clusters sticking to the surface of the carbon cloth, ranging from smaller, typically spherical islands with a size of ~10 nm to larger, elongated features stretching up to 200 nm in length, with a width of ~80 nm. EDX mappings were performed in the area depicted in Figure 2d and were conducted for the elements of carbon (C) (Figure 2e) and Pd (Figure 2f). These elemental mappings confirm that the islands detected on the carbon cloth surface consist of Pd, while the non-decorated areas show a significantly increased carbon content. These results agree well with the findings of the XPS studies. However, it cannot currently be determined whether these nano-sized islands are amorphous, or their content is too small to be detected by XRD.

### 3.5. Discussion on Growth Mechanisms

In the literature, Pd nanostructures have been synthesized using DC magnetron sputtering on templates. One characteristic example was reported by Arroyo-Ramírez et al. using poly(ethylene) oxide fibers [37]. In another study, Pantojas et al. used a combination of electrospinning for template synthesis and DC magnetron sputtering of Pd, with deposition times as low as 12.5 s [38]. In both cases fiber-like Pd nanostructures could be obtained after template removal. Even though the aforementioned approaches lead to hollow free-standing Pd shell nanostructures, no Pd-based nanocomposites were developed and characterized in these studies and no synergies are therefore expected between different phases with respect to chemical effects on the surface of these materials.

Furthermore, ion implantation techniques have been used to functionalize nanofibers and carbon fiber cloths [39,40]. Wu et al. [40] employed a combination of accelerated metal ions, including Ni, Fe and Co, followed by an etching step. This method created a porous structure with macropores of 70 nm or more in size. The etching step removed NPs from the surface but allowed for NPs to be retained within the pore structure. In contrast to the present work, the macropores obtained in [40] do not provide the functionalities associated with meso- and microporosity. 

The Pd decoration presented within this work can be described as the result of three-dimensional Volmer–Weber type island growth being dominant for the applied low deposition times, as evident from the morphology found via SEM investigations and the trend in chemical composition revealed via XPS studies. Due to the short deposition times, the coalescence of these growing islands is either not possible (for the smaller islands) or occurs only to a small extent (explaining the formation of elongated islands with lengths up to 200 nm). This growth mode is fostered by atomic shadowing, which cannot be overcome due to the lack of external heating and sufficient plasma heating during the low deposition times. The complex three-dimensional surface morphology of the ACC, which results in growth conditions comparable to glancing angle deposition under varying angles of incidence, may even exacerbate atomic shadowing effects, leading to the formation of the observed nano-sized features [41]. Additionally, local surface defects or impurities present on the ACC surface may offer beneficial growth conditions in the direction of the incoming flux of sputtered Pd atoms, leading to the formation of the observed elongated features [42].

In contrast, the development of a continuous Pd film would require significantly longer deposition times to achieve the coalescence of the islands and nano-rods, as demonstrated in fundamental works on thin film growth [25,43]. Growth of small Pd islands consisting of, on average, 60 atoms on Au(111) surfaces was reported by Stephenson et al. using Pd evaporation under low deposition rates [44]. To the best of the authors’ knowledge, no comparable surface morphology has been previously obtained for Pd deposited on carbon fibers using the DC magnetron sputtering processes. It should also be noted here that the applied DC magnetron sputtering technique offers excellent scalability to large area deposition [45], where the flexible ACC substrate even enables the high-throughput synthesis of such nanocomposites via roll-to-roll sputter deposition processes [46]. The flexible nature of the ACC substrate may even enable achieving a relatively homogeneous distribution of the Pd islands, when coating deposition is performed from both sides of the ACC substrate passing the magnetrons with varying bending radii and thus exposing shaded areas to the incoming flux of sputtered Pd atoms.

## 4. Conclusions

Within this work, we demonstrated for the first time that a laboratory-scale conventional direct current magnetron sputtering process could be effectively used for synthesizing Pd nano-sized islands, clusters or nanoparticles for the functionalization of activated carbon cloths, thus obtaining a metal–carbon nanocomposite material. The advantages of this synthesis method include the high purity of the deposited material due to the high purity of the metal target and the lack of chemical precursors, as is required for wet chemistry methods. Furthermore, more expensive and complex methods such as magnetron sputter inert gas condensation can be avoided. The suggested nanocomposite material concept could be potentially employed in emerging technologies, including cathodes for the oxygen reduction reaction in fuel cells, sorbents for materials-based hydrogen storage and sensing applications as well as detectors and sensors for biomolecules. In addition, sputter deposition methods are known for their scalability, enabling the large-scale synthesis of such complex Pd–carbon nanocomposites.

## Figures and Tables

**Table 1:** Comprehensive results of the gas sorption analysis deduced by N_2_ adsorption/desorption data recorded at 77 K for the *ACC* and *Pd-ACC-15s* samples.

Material	S_BET_ [m^2^/g]	S_micro_ [m^2^/g]	V_Gurvich_ [cm³/g]	V_micro_ [cm³/g]	d_50_ [nm]
ACC	1365	1279	0.57	0.50	0.77
Pd-ACC-15s	1202	1131	0.50	0.44	0.67
Change	−11.9%	−11.6%	−12.3%	−12%	−12.1%

**S_BET_**: Brunauer–Emmett–Teller (BET) specific surface area. **S_micro_**: micropore specific surface area calculated by the Carbon Black statistical thickness (t-plot) method. **V_Gurvich_**: total specific pore volume at P/P_0_ ~0.96 for pores smaller than 57 nm in width calculated by the single-point Gurvich rule. **V_micro_**: specific micropore volume calculated by the t-plot method. **d_50_**: the pore width corresponding to 50% of the cumulative specific pore volume, as determined by the quenched solid functional theory (QSDFT) method for slit-shaped pores.

## Data Availability

The data that support the findings of this study are available from the corresponding authors upon reasonable request.
